# The Effects of Postprandial Resistance Exercise on Blood Glucose and Lipids in Prediabetic, Beta-Thalassemia Major Patients

**DOI:** 10.3390/sports8050057

**Published:** 2020-04-26

**Authors:** Kalliopi Georgakouli, Alexandra Stamperna, Chariklia K. Deli, Niki Syrou, Dimitrios Draganidis, Ioannis G. Fatouros, Athanasios Z. Jamurtas

**Affiliations:** 1 Department of Physical Education and Sport Science, University of Thessaly, Karies, 42100 Trikala, Greece; kgeorgakouli@gmail.com (K.G.); alexstaberna@gmail.com (A.S.); delixar@pe.uth.gr (C.K.D.); nikisyrou@uth.gr (N.S.); dimidraganidis@gmail.com (D.D.); ifatouros@uth.gr (I.G.F.); 2 Department of Nutrition & Dietetics, University of Thessaly, Karies, 42100 Trikala, Greece

**Keywords:** haemoglobin, diabetes, fitness, cardiovascular health, nutrition

## Abstract

Insulin resistance and diabetes mellitus are common consequences of iron overload in the pancreas of beta-thalassemia major (BTM) patients. Moreover, postprandial blood glucose elevations are linked to major vascular complications. The purpose of this study was to investigate the effects of a bout of acute resistance exercise following breakfast consumption of glucose and fat on the metabolism in prediabetic, BTM patients. Six patients underwent two trials (exercise and control) following breakfast consumption (consisting of approximately 50% carbohydrates, 15% proteins, 35% fat), in a counterbalanced order, separated by at least three days. In an exercise trial, patients performed chest and leg presses (3 sets of 10 repetitions maximum/exercise), while in the control trial they rested. Blood samples were obtained in both trials at: pre-meal, 45 min post-meal (pre-exercise/control), post-exercise/control, 1 h post-exercise/control, 2 h post-exercise/control and 24 h post-exercise/control. Blood was analysed for glucose and lipids (total cholesterol, High Density Lipoprotein-cholesterol, Low Density Lipoprotein-cholesterol, triglycerides). Blood glucose levels increased significantly 45 min following breakfast consumption. Blood glucose and lipids did not differ between trials at the same time points. It seems that a single bout of resistance training is not sufficient to improve blood glucose and fat levels for the subsequent 24-h post-exercise period in prediabetic, BTM patients.

## 1. Introduction

Prediabetes is as a state of intermediate hyperglycaemia where a person has impaired fasting glucose, impaired glucose tolerance or a combination of the two [1]. Although glucose levels in prediabetes are not yet high enough for a diagnosis of Diabetes Mellitus (DM), prediabetes is associated with macrovascular and microvascular complications of DM, including nephropathy, small fibre neuropathy, retinopathy and coronary artery disease. Moreover, individuals with prediabetes are at high risk of developing DM [2].

Lifestyle interventions have been shown to reduce the risk of DM in adults with prediabetes, and they should be an essential part of the management of this condition. The main problem in glycaemic control is the peak of glucose 1–2 h after a meal, i.e., postprandial hyperglycaemia. Exercise increases contraction-mediated glucose uptake resulting in reduced postprandial hyperglycaemia and has been proposed as an effective way to improve glucose control in individuals with type 2 DM. Indeed, the timing of exercise relative to meal consumption may play a role in glycaemic control. The limited available data indicate that postprandial (consumption following dinner) exercise may be more beneficial than preprandial exercise in type 2 DM patients, and both aerobic and resistance training have been shown to be effective [3]. In addition, postprandial resistance exercise improves triglyceride levels, another risk factor for cardiovascular disease in type 2 DM [4]. Thus, postprandial resistance exercise may be an effective means of better glycaemic control and a lower risk of cardiovascular disease in individuals with an abnormal glucose metabolism. The optimal postprandial exercise timing and prescription are yet to be defined.

Beta-thalassemia major (BTM) is an inherited haemoglobin disorder that manifests within the first few months of life with ineffective erythropoiesis and chronic haemolytic anaemia, and frequent blood transfusions are required. There is no physiological mechanism to remove the excess iron load resulting from regular blood transfusions, while ineffective erythropoiesis increases intestinal iron absorption. Both processes induce iron accumulation in reticuloendothelial cells and parenchymal tissues that can cause progressive damage in multiple organs [5]. Iron accumulation in the pancreatic islets induces insulin resistance and reduced early insulin secretion, often resulting in DM in BTM patients [6]. Although the pathophysiological mechanisms leading to the development of DM are still unclear, it is most likely linked to the reversible oxidation and reduction of iron. This property renders iron potentially hazardous due to its ability to participate in the generation of reactive oxygen species [7], while pancreatic islets are susceptible to oxidative damage as they almost exclusively rely on the mitochondrial metabolism of glucose for glucose-induced insulin secretion and they also have a low antioxidant defence system [8]. Exercise is thought to provide various beneficial health effects in various metabolic disorders; however, research on the effects of exercise in BTM patients is non-existent. This could be due to the fact that BTM patients often manifest exercise intolerance and fatigue mediated by anaemia and iron-mediated cardiotoxicity [9].

This study was therefore designed to investigate whether postprandial resistance exercise can influence changes in glucose and lipid metabolism in prediabetic, BTM patients. Based on previous literature on the effects of postprandial exercise on health parameters in prediabetics, we hypothesized that an acute bout of resistance exercise 45 min following breakfast consumption would attenuate the blood glucose response and improve lipid profiles throughout the subsequent 24-h post-exercise period.

## 2. Materials and Methods

### 2.1. Participants

Six prediabetic, BTM patients (3 men, 3 women; age: 39.5 ± 4.6 years) volunteered to participate in this study. All of them had their doctor’s permission to participate, were informed about the study protocol, filled a medical history questionnaire and signed an informed consent form. Due to transfusions, these patients had suppressed their autologous hematopoiesis. Haemoglobin in BTM patients comes mainly from frequent transfusions and therefore has a normal O2 affinity. Procedures were in accordance with the 1975 Declaration of Helsinki (2000) and approval was obtained from the Institutional Review Board of the Department of Physical Education and Sport Science, University of Thessaly (protocol number 1076). Moreover, the study was registered at ClinicalTrials.gov as NCT03889977 [10].

Inclusion criteria:

- BTM patients requiring regular blood transfusions.
- Confirmed prediabetes with patients fulfilling one if the three following criteria: (a) impaired fasting glucose (IFG) (fasting plasma glucose (FPG) of 6.1–6.9 mmol/L), or (b) impaired glucose tolerance (IGT) (plasma glucose of 7.8–11.0 mmol/L, 2 h following ingestion of 75 g of oral glucose load) or (c) a combination of the two, based on a 2 h oral glucose tolerance test [1].
- Age group of 25 to 55.

Exclusion criteria:

- Hypertension.
- Injuries.
- Any other serious complications of BTM that could contraindicate participation to exercise.

Moreover, volunteers were instructed to avoid lifestyle changes and strenuous or unusual physical activity for at least two days before each visit to the laboratory.

### 2.2. Experimental Design

All participants reported to the laboratory in the morning (9:00–10:00 a.m.) for physiological measurements (blood pressure, resting heart rate, body composition analysis through DEXA test) and determination of one repetition maximum (1RM). Moreover, they were asked to record their diet for two days before their next visit and followed the same diet before the third and last visit.

In a randomized within-subject design, participants underwent two trials (exercise, ExT and control, CoT) 45 min following breakfast consumption (consisting of approximately 50% carbohydrates, 15% proteins, 35% fat), in a counterbalanced order, separated by at least three days.

In each one of the experimental trials, participants reported to the laboratory in the morning (9:00–10:00 a.m.) after overnight fasting (~10 h) and a blood sample was drawn. Then they were provided with a standard breakfast that they had to consume in 10 min. Forty-five min later, another blood sample was drawn. In the ExT, the participants initially performed a warm-up for 5–10 min consisting of 8–10 repetitions using a light weight. Then they performed chest and leg press exercises (3 sets of 10 repetitions at 70% of their 1RM, each), and they finished the workout by performing stretching exercises for approximately 5 min of the two major muscle groups used during the workout. In the CoT, participants rested for the same duration. Blood samples were obtained immediately after ExT and CoT, as well as 1 h, 2 h and 24 h following each trial. Water consumption throughout trials was ad libitum.

### 2.3. Anthropometric and Physiological Characteristics

Body height was measured with a precision of 0.1 cm and body weight with a precision of 0.1 kg (Beam Balance, Seca, Birmingham, UK), with the participants lightly dressed and barefoot. Body fat percentage was estimated by dual-energy X-ray absorptiometry (DEXA) (Lunar DPX NT, GE Healthcare, Chalfont St. Giles, Buckinghamshire, UK). Blood pressure (BP) was measured with a manual sphygmomanometer (FC-101 Aneroid Sphygmomanometer; Focal Corporation, Kashiwa, Japan) after 5 min of seated rest.

### 2.4. Blood Collection and Handling

In total, blood samples were obtained from a forearm vein with participants in a seated position at the following time-points: pre-meal, 45 min post-meal (pre-ExT/CoT), immediately post- ExT/CoT, 1 h post-ExT/CoT, 2 h post-ExT/CoT, 24 h post- ExT/CoT. Blood samples were obtained after 10 min rest, except for immediately post-ExT, where blood was obtained immediately following exercise. Blood was immediately transferred to a tube containing Ethylene diamine tetraacetic acid (EDTA) and centrifuged in order to obtain the plasma which was stored in aliquots at −80 °C until the day of analysis. Plasma preparation has been described elsewhere [11].

### 2.5. Blood Analysis

Samples underwent only one freeze-thaw cycle and each parameter was measured in duplicates. Plasma glucose and lipids (total cholesterol, HDL-cholesterol, LDL-cholesterol, triglycerides) were determined using a biochemical analyser (Clinical Chemistry Analyzer Z 1145; Zafiropoulos Diagnostica S.A., Koropi, Greece). LDL-cholesterol was calculated using the Friedewald equation [12].

### 2.6. Statistical Analysis

Preliminary power analysis performed with G*Power [13] showed that the minimum required sample size was 6 (with a probability error of 0.05, a statistical power of 80% and an effect size of 0.5).

Normality was checked using the Shapiro–Wilk test. Since some variables did not follow normal distribution, nonparametric statistics were used for the analyses. The Friedman analysis of variance by ranks test was performed to determine the time effects, accompanied by the Wilcoxon signed-rank test to perform pairwise comparisons. Differences between trials were examined using the Mann–Whitney U test (U values). Differences between genders for areas under the curve (AUC) and triglycerides was assessed by a 2 × 2 (gender by condition) ANOVA. The level of statistical significance was set at *p* < 0.05. Data are presented as means ± SD. Statistical analysis was conducted with IBM SPSS Version 19.0 (IBM Corp., Armonk, NY, USA).

Moreover, the incremental areas under the curves (AUCs) were measured for plasma glucose and triglycerides with GraphPad Prism version 5.0 (GraphPad Software, San Diego, CA, USA). Differences in AUCs between trials were examined using the Mann–Whitney U test (U values).

## 3. Results

### 3.1. Anthropometric and Physiological Characteristics

Anthropometric and physiological characteristics of the participants did not differ between the ExT and CoT (Table 1). The mean body mass index (BMI) indicated that men were overweight and women were normal weight. According to the International Diabetes Federation, the ethnic group specific cut-point for waist circumference (WC) is 94 cm and 80 cm for European men and women, respectively [14]. WC of the participants in the present study exceeded this cut-point, indicating central obesity.

### 3.2. Metabolic Parameters

**Figure 1:**
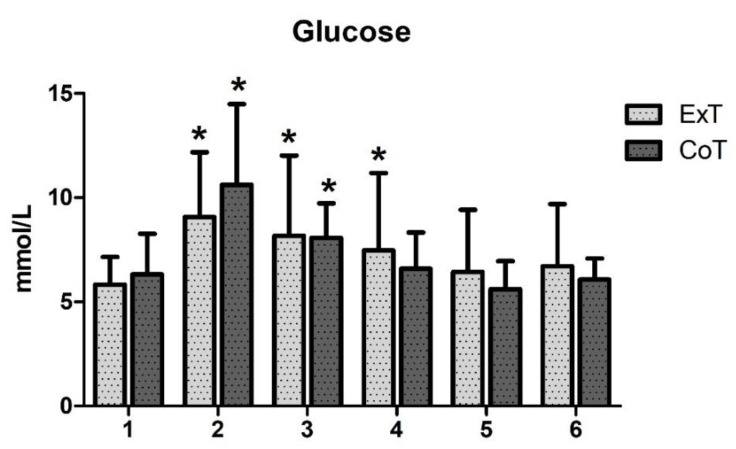
Changes in plasma glucose levels (mmol/L) following exercise (ExT) and control trial (CoT). Time points: (1) pre-meal; (2) 45 min post-meal (pre-exercise/control); (3) immediately post-exercise/control; (4) 1 hr post-exercise/control; (5) 2 h post-exercise/control; (6) 24 h post-exercise/control. *Significant difference from (1) at the same trial.

**Figure 2:**
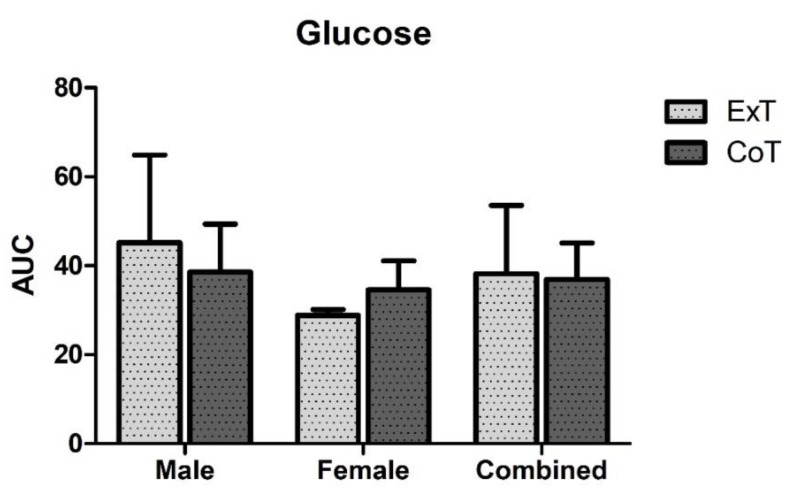
Glucose area under the curve (AUC) in mmol/L × 24h in exercise (ExT) and control trial (CoT).

In ExT, plasma glucose levels increased 45 min following breakfast consumption (%z = −2.20; *p* = 0.028), immediately following exercise (%z = −1.99; *p* = 0.046) and 1 h following exercise (%z = −1.99; *p* = 0.046) compared to baseline levels (before breakfast consumption). In CoT, plasma glucose levels were increased 45 min following breakfast consumption (%z = −2.20; *p* = 0.028) and immediately following rest (%z = −2.02; *p* = 0.043) compared to baseline levels (before breakfast consumption). Moreover, pairwise comparisons showed that there was no significant difference in glucose levels at any time point between trials (Figure 1). Table 2 indicates time related changes with significant increases observed at 45 min post-meal following the exercise and control trials, immediately post-exercise/control and 1 h post-exercise. Moreover, no difference in glucose area under the curve (AUC) between trials or gender was observed (Figure 2).

**Figure 3:**
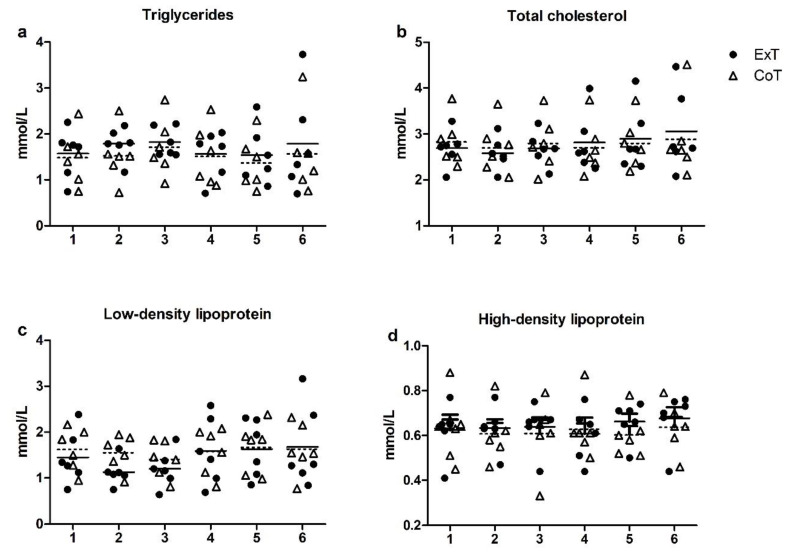
Changes in plasma lipid levels following exercise (ExT) and control trial (CoT): (**a**) Triglycerides; (**b**) Total cholesterol; (**c**) LDL: Low Density Lipoprotein; (**d**) HDL: High Density Lipoprotein. Time points: (1) pre-meal; (2) 45 min post-meal (pre-exercise/control); (3) immediately post-exercise/control; (4) 1 h post-exercise/control; (5) 2 h post-exercise/control; (6) 24 h post-exercise/control. Dotted lines represent the mean values of the Control trial.

**Figure 4:**
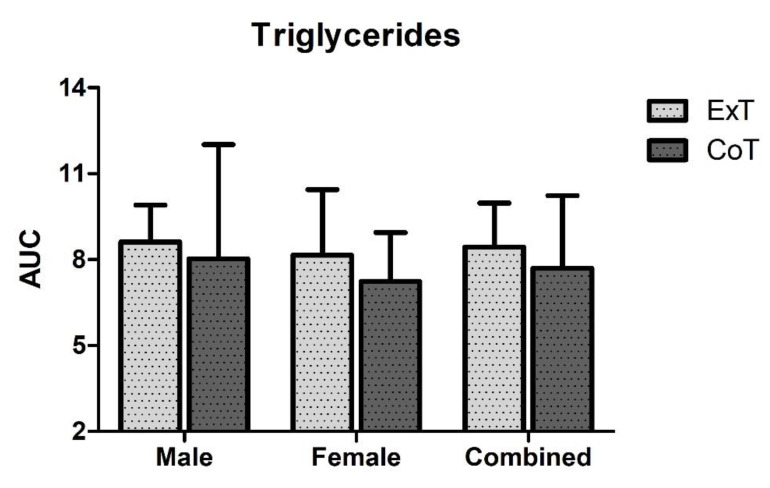
Triglycerides area under the curve (AUC) in mmol/L × 24h in exercise (ExT) and control trial (CoT).

Triglycerides, total cholesterol, HDL and LDL levels did not change at any time point and were similar in both conditions (Figure 3a–d). Moreover, no difference in triglycerides AUC between trials or gender was observed (Figure 4).

## 4. Discussion

The main aim of this study was to examine whether postprandial resistance exercise can influence changes in blood glucose in prediabetic, BTM patients. Supplementary analyses of blood lipids were performed as these parameters are associated with glucose metabolism, DM and cardiovascular disease. To the authors’ knowledge, this is the first study to test this hypothesis in this clinical population. Our findings suggest that an acute bout of postprandial (post-breakfast) resistance exercise is not a sufficient stimulus to (i) attenuate the blood glucose response, and (ii) change the lipids profile, throughout the subsequent 24-h post-exercise period.

Although research on the effects of resistance training on glycaemic control in type 2 DM patients is scarce, there is some data suggesting that this modality could be beneficial [15]. Moreover, the positive effects of a single session of aerobic exercise (50% maximum workload capacity) and a single session of resistance exercise (75% 1RM) on the 24-h average blood glucose levels and the 24-h prevalence of hyperglycaemia were found to be similar [16]. In the present study, we did not observe any change in blood glucose levels throughout the subsequent 24-h post-exercise period. It is possible that prediabetic, BTM patients do not have similar responses to those of type 2 DM patients as the underlining pathophysiology could lead to different results. Moreover, the high level of SD observed could be explained by the small sample. The population examined in our study has very unique characteristics and many factors (i.e., medication, frequency of blood transfusion, comorbidities) can lead to different responses. Moreover, SD was higher in Ext at time points 2–6, indicating that these unique characteristics may have an important role in blood glucose responses to exercise for up to 24-h. Besides, it has been reported that BTM patients often manifest exercise intolerance and fatigue mediated by anaemia and iron-mediated cardiotoxicity [9]. Thus more research comparing the effects of resistance exercise in prediabetic, BTM patients and DM patients is warranted.

The timing of exercise relative to meal consumption may also play a role in glycaemic control. The limited available data indicate that postprandial exercise may be more beneficial than preprandial exercise in type 2 DM patients [3]. Postprandial resistance exercise causes a greater reduction in glucose incremental area under the curve (iAUC) (reduction by 30%) compared to preprandial resistance exercise (reduction by 18%) [4]. In addition, postprandial resistance exercise improves triglyceride levels, another risk factor for cardiovascular disease in type 2 DM [4]. Thus, postprandial resistance exercise may be an effective means of better glycaemic control and lower risk of cardiovascular disease in individuals with abnormal glucose metabolism. Based on this, Borror et al. [3] proposed that resistance exercise should be performed following the largest meal of the day, 2 to 3 non-consecutive days per week, at intensities varying between 50% and 80% of 1RM, working the major muscle groups (1–4 sets of 8–15 repetitions/exercise) [17,18]. In the present study, we used an acute resistance exercise protocol at an intensity of 70% of 1RM and we found no change in blood glucose levels, glucose AUC, lipid levels or triglycerides AUC throughout the 24-h post-exercise period. The limited available data suggest that resistance exercise could have positive effects that are associated with increases in lean muscle mass and type II fibre type recruitment [15]. However, all aforementioned studies refer to acute postprandial exercise studies and it needs to be stated that it is of great importance to perform exercise training interventions to assess the long-term effects of exercise on not only the acute hyperglycaemia, but the long-term glycaemia control as well. Previous reports indicate that glycaemia in type 2 DM males and females is different with males exhibiting higher impaired fasting glycaemia and women impaired glucose tolerance [19,20]. The results from this study do not coincide with the aforementioned reports since there were no differences between males and females neither at rest nor following the glycaemic load (AUC) at rest and after exercise. However, it needs to be stated here that the sample size for males (n = 3) and females (n = 3) is rather small, this constitutes a limitation of the study and concrete conclusions should be made with caution.

Postprandial hypertriglyceridemia has also been linked to increased risk of cardiovascular disease [21]. In this study, blood lipids (triglycerides, total cholesterol, HDL and LDL) did not change at any time point and were similar in both trials. This was also evident in the lack of difference in triglycerides AUC between trials. This may be explained by the fact that the breakfast provided was low in saturated fat. A meal high in saturated fat increases blood triglyceride levels as well as indices of oxidative stress and inflammation, resulting in a worsening of endothelial dysfunction, vasoconstriction and systolic blood pressure [22,23]. Therefore, exercise following lunch or dinner could be more beneficial in terms of cardiovascular health.

Patients with abnormal glucose levels are often diagnosed with Metabolic Syndrome (MS), a multiple set of risk factors that confer an additional cardiovascular risk [24]. In this study, it was shown that men were overweight and women were of normal weight according to BMI, and that both genders had central obesity according to the WC cut-point [14]. The participants were also prediabetic and had low HDL levels, meeting the criteria for metabolic syndrome set by the International Diabetes Federation. As already mentioned, BTM causes various complications due to anaemia and iron overload, and the presence of MS is an additional cardiovascular risk, rendering BTM patients a clinical population with unique characteristics. Therefore, BTM patients may not respond to exercise in a typical manner and long-term interventions are required.

In conclusion, postprandial resistance exercise, especially following the largest meal of the day, could be an effective means of glycaemic control and could lower the risk of cardiovascular disease in DM patients [3]. The results of the present study do not indicate that a bout of acute resistance exercise 45 min following breakfast is a sufficient stimulus to improve blood glucose lipid levels throughout the subsequent 24-h post-exercise period. Future studies comparing the acute and chronic effects of resistance exercise in prediabetic, BTM patients and DM patients are needed.

## Figures and Tables

**Table 1:** Anthropometric and physiological characteristics before exercise (ExT) and control trial (CoT).

Variable	ExT	CoT
BM (kg)	66.0 ± 16.6	65.5 ± 16.2
BMI (kg/m^2^)	24.2 ± 5.4 (men: 25.7 ± 7.2; women: 22.6 ± 3.8)	24.0 ± 5.2 (men: 25.6 ± 6.8; women: 22.4 ± 3.7)
%BF	37.6 ± 5.1 (men: 25.7; women: 22.6)	37.6 ± 5.1 (men: 25.7; women: 22.6)
WC (cm)	93.5 ± 12.2 (men: 101.7 ± 9.1; women: 85.3 ± 9.5)	93.0 ± 11.6 (men: 101.0 ± 7.9; women: 85.0 ± 9.0)
HC (cm)	97.5 ± 8.2 (men: 100.3 ± 8.5; women: 94.7 ± 8.5)	97.5 ± 8.2 (men: 100.3 ± 8.5; women: 94.7 ± 8.5)
WHR	0.96 ± 0.07 (men: 1.01 ± 0.03; women: 0.90 ± 0.02)	0.95 ± 0.06 (men: 1.00 ± 0.03; women: 0.90 ± 0.01)
RHR	79.0 ± 8.6	77.5 ± 9.2
SBP (mmHg)	104.5 ± 9.7	103.7 ± 10.3
DBP (mmHg)	67.5 ± 7.6	67.5 ± 7.6

BM: Body Mass; BMI: Body Mass Index; %BF: Body Fat percentage; WC: Waist Circumference; HC: Hip Circumference; WHR: Waist to Hip Ratio; RHR: Resting Heart Rate; SBP: Systolic Blood Pressure; DPB: Diastolic Blood Pressure.

**Table 2:** Changes in plasma glucose (mmol/L) levels (mean ± SD) following exercise (ExT) and control trial (CoT). Time points: (1) pre-meal; (2) 45 min post-meal (pre-exercise/control); (3) immediately post-exercise/control; (4) 1 h post-exercise/control; (5) 2 h post-exercise/control; (6) 24 h post-exercise/control. * Significant difference from (1) at the same trial.

Time point	ExT	CoT
1	5.83 ± 1.33	6.32 ± 1.95
2	9.07 ± 3.11 *	10.62 ± 3.87 *
3	8.17 ± 3.85 *	8.07 ± 1.66 *
4	7.47 ± 3.71 *	6.60 ± 1.73
5	6.43 ± 2.99	5.61 ± 1.35
6	6.71 ± 2.99	6.07 ± 1.01
